# The Influence of NaCl and Glucose Content on Growth and Ochratoxin A Production by *Aspergillus ochraceus*, *Aspergillus carbonarius* and *Penicillium nordicum*

**DOI:** 10.3390/toxins12080515

**Published:** 2020-08-12

**Authors:** Yan Wang, Hao Yan, Jing Neng, Jian Gao, Bolei Yang, Yang Liu

**Affiliations:** 1 College of Food science and Technology, Zhejiang University of Technology, Hangzhou 310014, China; wangyan062006@zjut.edu.cn (Y.W.); nengjing@zjut.edu.cn (J.N.); 2 Zhejiang Provincial Center for Disease Control and Prevention, Hangzhou 310051, China; 3 Institute of Food Science and Technology, Chinese Academy of Agricultural Sciences, Beijing 100193, China; gaojian_2020@126.com (J.G.); yangbolei@caas.cn (B.Y.); 4 School of Food Science and Engineering, Foshan University, Foshan 528231, China

**Keywords:** ochratoxin A, *A. ochraceus*, *A. carbonarius*, *P. nordicum*, NaCl-riched, high-sugar

## Abstract

Ochratoxin A (OTA) is a nephrotoxic mycotoxin, which deserves particular attention for its widespread contamination of a variety of food and feed. *Aspergillus ochraceus*, *Aspergillus carbonarius*, and *Penicillium nordicum* are an important source of OTA in three different kinds of food commodities, including cereals, grape and dried fruit products, and dry-cured meat products. Deeper knowledge of OTA production and mycelium growth related to the high-sugar or NaCl-rich environments was gained in this manuscript. *A. ochraceus* and *P. nordicum* were likely to have greater growth rates in medium supplied with certain concentrations of NaCl (0–80 g/L), and the colony diameter was the largest at the salt content of 40 g/L. *P. nordicum* was more suitable to grow in NaCl-riched medium, the OTA production was increased to 316 ppb from 77 ppb when 20 g/L NaCl was added. The capability of OTA production was inhibited when salt content was 40 g/L and 60 g/L in *A. ochraceus* and *P. nordicum*, respectively. As the glucose content increased to 250 g/L, the capacity of mycelium growth and sporulation was increased significantly in *A. ochraceus* and *A. carbonarius*. *A. carbonarius* was more suitable to grow in high-sugar grape products. OTA production was significantly promoted with an added 100 g/L glucose in *A. carbonarius*. OTA production was inhibited when glucose content was 150 g/L and in 200 g/L in *A. ochraceus* and *A. carbonarius*, respectively. NaCl and glucose have an effect on fungal growth and OTA production, and the activation of biosynthetic genes of OtaA. These results would allow designing new strategies to prevent OTA accumulation on sugar or NaCl-riched foodstuffs and achieve the objective to manufacture cereals, dried vine fruits and dry-cured ham, free of OTA.

## 1. Introduction

Ochratoxin A (OTA) is a nephrotoxic mycotoxin, which deserves particular attention as it is a widespread toxin in various contaminated foods and types of feed. Because of the temperature and humidity preferences, ochratoxigenic *Aspergillus* and *Penicillium* occur mainly on food commodities like cereal (*Aspergillus ochraceus*, *Aspergillus westerdijkiae*, *Penicillium verrucosum*), coffee (*Aspergillus steynii*, *Aspergillus sclerotioniger*), grapes, wine and dried fruits (*Aspergillus carbonarius*, *Aspergillus niger*, *A. ochraceus*), cheese and dry-cured meat products (*Penicillium nordicum*) [1,2]. OTA is inevitably ingested by human beings and animals as a result. A great deal of animal or cell experiments have reported that the OTA exposure can cause nephrotoxicity, hepatotoxicity, immunotoxicity, genotoxicity and other toxicological effects [3]. OTA was classified as a group 2B carcinogen by the International Agency for Research on Cancer (IARC) in 1993.

*A. ochraceus*, *A. carbonarius* and *P. nordicum* are believed to be important sources of OTA in food commodities [2]. Ochratoxin A production is in relation to water activity and temperature [4]. *A. ochraceus* and closely related species grow under moderate temperatures at low water activities [5]. They are mostly associated with dried and stored foods, especially cereal and coffee. Black *Aspergilli*, mainly *A. carbonarius*, are considered as the primary source of OTA on grapes and similar fruits produced on the berries during the growing season, mainly from maturing to ripening in sunlight at high temperatures [6,7,8]. In the traditional meat products with long maturity periods, especially in fermented meat products, toxigenic moulds are mainly comprised of *Aspergillus* and *Penicillium* spp., which often overgrow [9]. *P. nordicum* is adapted to NaCl rich environments like salt rich dry-cured meat products, cheeses or even salines [10]. On the contrary, *A. carbonarius* frequently exists in sugar rich substrates like grapes and grape juices, which is another environment with high concentrations of solutes different from NaCl rich environments [11].

The decrease in water activity ensures the microbial stability and safety of food products. High concentrations of solutes, e.g., in NaCl rich substrates like ham and cheeses, or in sugar rich substrates like grapes and grape juices, not only decrease the water activity of the substrate, but also provoke osmotic stress, affecting the adaptation of this specific fungal population and toxin production [10,11,12]. OTA biosynthesis is in charged of a gene cluster, which contains four highly conserved biosynthetic genes (otaA-D) and a bZIP transcription factor (otaR1). A pathway-specific regulator, OtaR1, controls OTA production by regulating the four OTA biosynthetic genes [13]. Polyketide synthase OtaA is the first limited enzyme in the synthesis of OTA. Halogenation by the halogenase OtaD is the last step of the synthesis of OTA, and the chloride ion is related to substitution reaction [13,14]. The biosynthesis of ochratoxin A by *Penicillium* is considered as one mechanism for adaptation to NaCl rich foods [15].

Thus, further studies are required to fully understand the advantageous adaptation of *A. ochraceus*, *A. carbonarius*, and *P. nordicum* in high concentrations of solutes, and their ability to produce OTA. The aim of this study was to adapt different concentrations of salt solution and sugar solution to determine simultaneously the effects on the growth and OTA production and the high concentration solution on the OTA biosynthesis gene expression in *A. ochraceus*, *A. carbonarius*, and *P. nordicum*. The aim of this work was to evaluate the influence of exogenous NaCl or glucose on mycelial growth rate and expression levels of OTA biosynthetic genes in relation to OTA production; the results would allow designing new strategies to prevent OTA accumulation on ham, cheese, grape juice and so on, achieving the goal to manufacture food free of OTA.

## 2. Results

### 2.1. The Colony Morphology Are Affected by High Concentration of NaCl or Glucose

**Figure 1:**
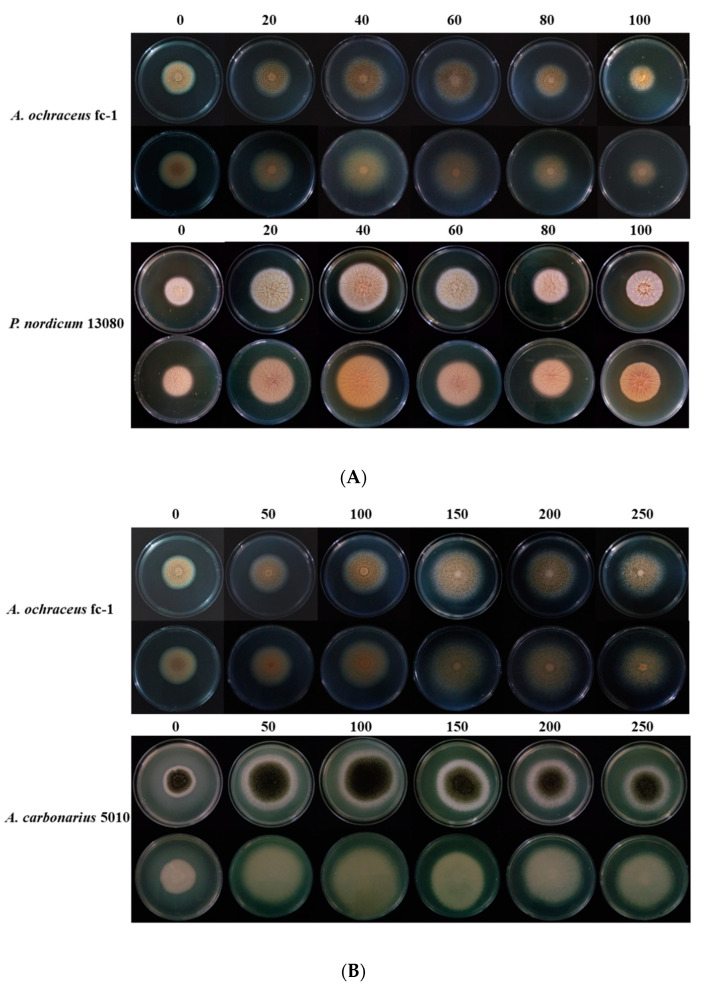
The colony of *A. ochraceus* fc-1, *A. carbonarius* 5010, *P. nordicum* 13080 after 4 days of incubation at 28 °C: (**A**) Colonies of *A. ochraceus fc-1* grown on 0, 20, 40, 60, 80, 100 g/L NaCl-supplemented Potato Dextrose Agar (PDA) plates. Colonies of *P. nordicum 13080* grown on NaCl-supplemented Malt Extract Agar (MEA) plates. (**B**) Colonies of *A. ochraceus fc-1* grown on 0, 50, 100, 150, 200, 250 g/L glucose-supplemented PDA plates. Colonies of *A. carbonarius 5010* grown on glucose-supplemented Yeast Extract Sucrose Agar (YES) plates.

To investigate the influence of high osmotic conditions on growth and spore production, different concentrations of NaCl (0, 20, 40, 60, 80, 100 g/L) or glucose (0, 50, 100, 150, 200, 250 g/L) were added into medium. The colony morphology was observed for 24 h to 108 h after incubation. There was no significant change in colony morphology and colony color at high osmotic conditions (Figure 1).

**Figure 2:**
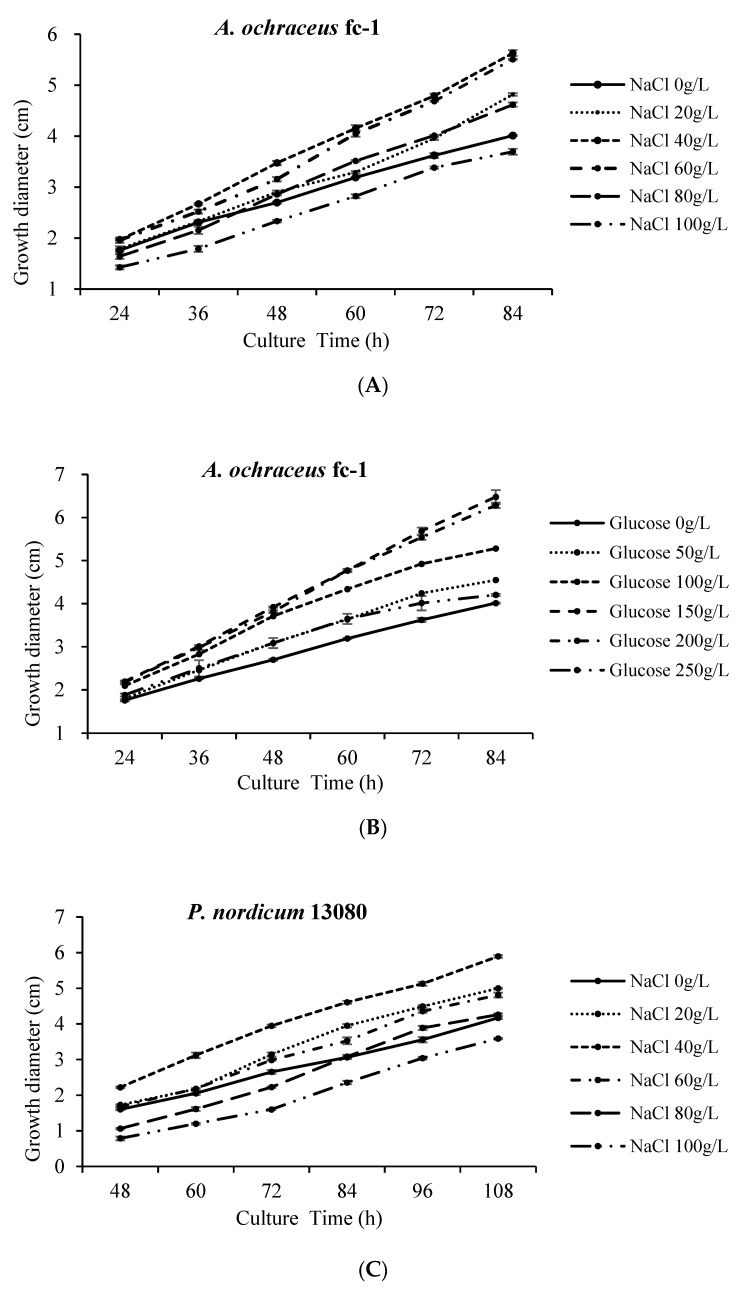

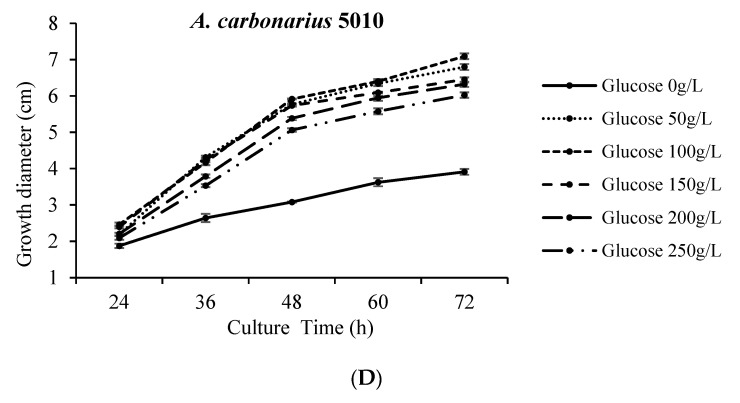
The colony diameter of *A. ochraceus* fc-1, *A. carbonarius* 5010, *P. nordicum* 13080 after 4 days of incubation at 28 °C: (**A**) The colony diameter of *A. ochraceus* fc-1grown on NaCl-supplemented PDA plates at different culture times; (**B**) The colony diameter of *A. ochraceus* fc-1grown on glucose-supplemented PDA plates at different culture times; (**C**) The colony diameter of *P. nordicum* 13080 grown on NaCl-supplemented MEA plates at different culture times; (**D**) The colony diameter of *A. carbonarius* 5010 grown on glucose-supplemented YES plates at different culture times.

The mycelial growth rates are clearly affected by high osmotic conditions (Figure 1). With the increase in NaCl concentration, the growth of hyphae showed a trend of first strengthening and then weakening. Low concentrations of NaCl promoted the growth of *A. ochraceus* fc-1 (Figure 2A). When 40 g/L NaCl was added, the diameter of the colony was 40.3% larger than that of the control group without NaCl. When 80 g/L NaCl was added, the growth rate of mycelia was weakened during the first two days of culture. When 100 g/L NaCl was added, the diameter of the colony was 7.9% smaller than that of the control group without NaCl (Figure 2A).

With the addition of glucose at 0–200 g/L, the growth of hyphae was gradually accelerated (Figure 2B). With the addition of 150 g/L glucose, the growth rate of hyphae was the highest. The colony diameter was 61.4% larger than that of the control group without glucose. With the addition of 250 g/L glucose, the growth rate of mycelia was weakened, while the colony diameter was still 4.8% larger than that of the control group (Figure 2B).

The effect of NaCl on *P. nordicum* 13080 is consistent with *A. ochraceus* fc-1 (Figure 2C). Low concentrations of NaCl promoted the growth of *P. nordicum* 13080. When 40 g/L NaCl was added, the diameter of the colony was 41.4% larger than that of the control group without NaCl. When 80 g/L NaCl was added, the growth rate of mycelia was reduced during the first three days of the culture; however, the growth rate of mycelia was still 2.2% larger than that of the control on the fifth day. When 100 g/L NaCl was added, the diameter of the colony was 13.9% smaller than that of the control group without NaCl (Figure 2C).

Glucose promoted the mycelial growth of *A. carbonarius* 5010 significantly more than that of *A. ochraceus* fc-1 (Figure 2D). Among the addition of glucose at 0–250 g/L, the growth of hyphae was significantly accelerated compared to the control without glucose. With the addition of 100 g/L glucose, the growth rate of hyphae was the highest. The colony diameter was 81.4% larger than that of the control group without glucose (Figure 2D).

### 2.2. The Spore Production Are Affected by High Concentration of NaCl or Glucose

**Figure 3:**
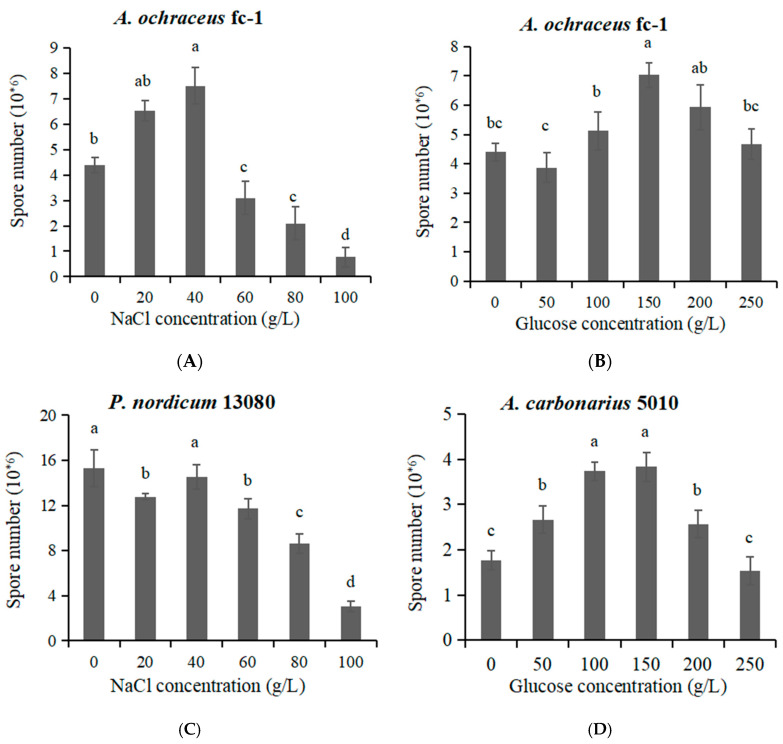
Spore production of *A. ochraceus* fc-1, *A. carbonarius* 5010 and *P. nordicum* 13080. (**A**) Spores were collected from *A. ochraceus* fc-1grown on 0, 20, 40, 60, 80, 100 g/L NaCl-supplemented PDA plates; (**B**) Spores were collected from *A. ochraceus* fc-1grown on 0, 50, 100, 150, 200, 250 g/L glucose-supplemented PDA plates; (**C**) Spores were collected from *P. nordicum* 13080 grown on NaCl-supplemented MEA plates; (**D**) Spores were collected from *A. carbonarius* 5010 grown on glucose-supplemented YES plates. Different letters indicate a significant difference between the corresponding values (*p* < 0.05).

When the concentration range was 0–40 g/L NaCl for the culture of *A. ochraceus* fc-1, the capacity of spore producing was improved (Figure 3A). When the concentration range was greater than 60 g/L NaCl, the spore-producing capacity was reduced significantly. When 80 g/L NaCl was added, the spore production was 61.4% of the control group without NaCl (Figure 3A). With the addition of glucose at the concentration range of 0–250 g/L, the spore-producing capacity could be improved to different degrees (Figure 3B). When 150 g/L glucose was added, the spore-producing capacity was strongest and the spore production was 70.5% larger than that of the control group without glucose (Figure 3B).

With the addition of NaCl, the spore-producing capacity of the *P. nordicum* 13080 decreased to different degrees. When 80 g/L NaCl was added, the spore production was 42.6% lower than that of the control group without NaCl (Figure 3C).

With the addition of glucose at the concentration range of 0–200 g/L, the spore-producing capacity could be improved to different degrees (Figure 3D). When 250 g/L glucose was added, the spore production was slightly lower than that of the control. With the addition of 150 g/L glucose, the spore-producing capacity was the strongest. The spore production was 2.17 times larger than that of the control group without glucose (Figure 3D).

### 2.3. The OTA Production Effected by High Concentration of NaCl or Glucose

**Figure 4:**
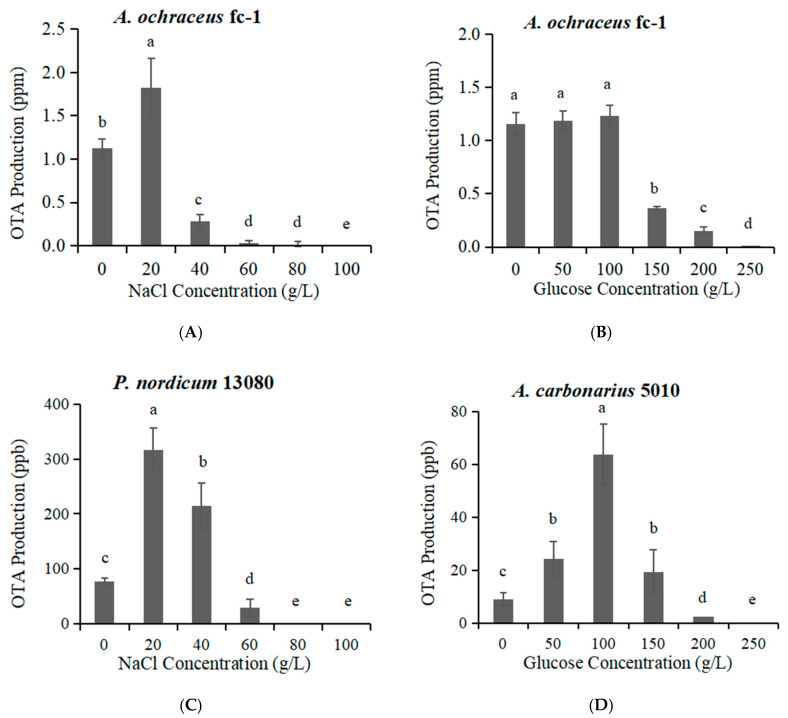
The Ochratoxin A (OTA) production of *A. ochraceus* fc-1, *A. carbonarius* 5010 and *P. nordicum* 13080 after 5 days of incubation. (**A**) The OTA production of *A. ochraceus* fc-1grown on NaCl-supplemented PDA plates; (**B**) The OTA production of *A. ochraceus* fc-1grown on glucose-supplemented PDA plates; (**C**) The OTA production of *P. nordicum* 13080 grown on NaCl-supplemented MEA plates; (**D**) The OTA production of *A. carbonarius* 5010 grown on glucose-supplemented YES plates. Different letters indicate a significant difference between the corresponding values (*p* < 0.05).

Low-concentration NaCl had a promoting effect on the capacity of OTA production by *A. ochraceus* fc-1 (Figure 4A). When 20 g/L NaCl was added, OTA production was significantly increased, which was 72.7% more than that of the control group in the absence of NaCl. When 40 g/L NaCl was added, OTA production decreased, which was 73.6% lower than that of the control group without NaCl. When 60 g/L NaCl was added, trace OTA could be detected; OTA could not be detected at 80 g/L NaCl or higher (Figure 4A).

Low-concentration glucose (0–100 g/L) had almost no effect on the capacity of OTA production (Figure 4B). With the addition of 150 g/L glucose, the OTA yield decreased, which was 65.0% lower than that of the control group without glucose. With the addition of 250 g/L glucose, the OTA yield was very low, 0.5% of the control group in absence of glucose (Figure 4B).

Similar with *A. ochraceus* fc-1, the capacity of OTA production by *P. nordicum* 13080 was increased and then decreased as the NaCl concentration increased (Figure 4C). When 20 g/L NaCl was added, the capacity of OTA production was 4.1 times higher than that of the control group. Compared to *A. ochraceus* fc-1, the capacity of OTA production by *P. nordicum* 13080 was still increased, and 2.8 times higher than that of the control group, when the addition was of 40 g/L NaCl. OTA could not be detected at 80 g/L NaCl or higher (Figure 4C).

Different from the tendency of OTA production in *A. ochraceus* fc-1, the capacity of OTA production by *A. carbonarius* 5010 was increased and then decreased as the glucose concentration increased (Figure 4D). With the addition of glucose at 0–150 g/L, the OTA production was higher than that of control group. When 100 g/L glucose was added, the capacity of OTA production was 7.1 times higher than that of control group. When 200 g/L glucose was added, trace OTA could be detected; OTA could not be detected at 250 g/L glucose (Figure 4D).

### 2.4. Relationship between Water Activity (Ionic Concentration) and OTA Production

With the addition of NaCl or glucose, the water activity decreased gradually, and the water activity was negatively correlated with ion concentration. The water activity of the medium with a salinity of 0, 20, 40, 60, 80 and 100 g/L was 0.991, 0.975, 0.962, 0.950, 0.934 and 0.929, respectively. The water activity of the medium containing 0, 50, 100, 150, 200 and 250 g/L glucose was 0.991, 0.992, 0.988, 0.981, 0.971 and 0.965, respectively. Pearson’s correlation coefficient (R^2^) was used to assess the correlation between water activity and ion concentration. A high Pearson’s correlation coefficient R^2^ = 0.993 (*p* < 0.01) and R^2^ = 0.956 (*p* < 0.01) was found between water activity and ionic concentration (NaCl, glucose), indicating that the water activity was closely correlated with ionic concentration.

Further, the relationship of OTA production with ionic concentration, water activity, colony diameter, and spore number in *A. ochraceus*, *A. carbonarius*, and *P. nordicum* were analyzed. The correlation was only observed between glucose concentration and spore number in *A. ochraceus* (R^2^ = 0.993, *p* < 0.05), between NaCl concentration and spore number in *P. nordicum* (R^2^ = 0.899, *p* < 0.05). The ionic concentration was not significantly associated with fungi growth and OTA production.

### 2.5. The Expression of OTA Biosynthetic Genes Effected by the Addition of NaCl or Glucose

**Figure 5:**
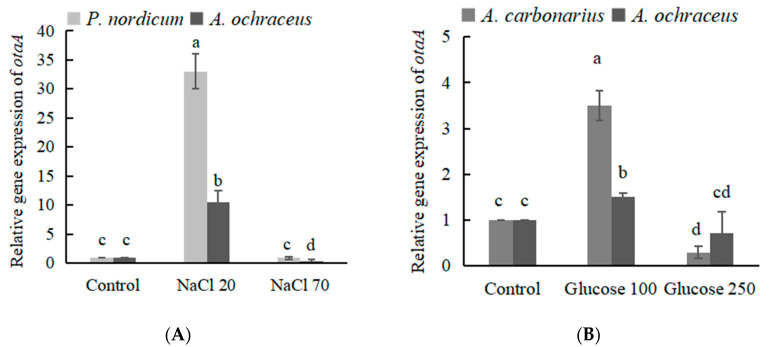
The relative expression level of OTA biosynthetic genes *AootaA* in *A. ochraceus* fc-1, *AcotaA* in *A. carbonarius* 5010 and *PnotaA* in *P. nordicum* 13080 at high osmotic conditions after 5 days of incubation. (**A**) The relative expression level of *otaA* on NaCl-supplemented medium; (**B**) The relative expression level of *otaA* on glucose-supplemented medium. Different letters indicate a significant difference between the corresponding values (*p* < 0.05).

The expression of OTA biosynthetic genes (*otaA-D*) and regulatory genes (*otaR1*) were detected by qRT-PCR. OTA biosynthesis begins with a polyketide synthase, OtaA, utilizing acetyl-CoA and malonyl-CoA to synthesize 7-methylmellein, which is a speed-limiting step of the synthesis of OTA. The transcriptional expression level of *AootaA* was increased 10.5 times in *A. ochraceus* at mediun supplied with 20 g/L NaCl, the *PnotaA* gene expression was up regulated 33.0 times in *P. nordicum* at mediun supplied with 20 g/L NaCl (Figure 5A). When 70 g/L NaCl was added, the expression level of *Aoota A* was 0.4 times lower than that of the control group without NaCl (Figure 5A). With the addition of 100 g/L, 250 g/L glucose, the expression level of *AootaA* was 1.5 and 1.7 times that of the control group without glucose, respectively (Figure 5B). The *AcotaA* gene expression was up regulated 3.5 times in *A. carbonarius* at mediun supplied with 100 g/L glucose, and down regulated 0.3 times in medium supplied with 250 g/L glucose.

**Figure 6:**
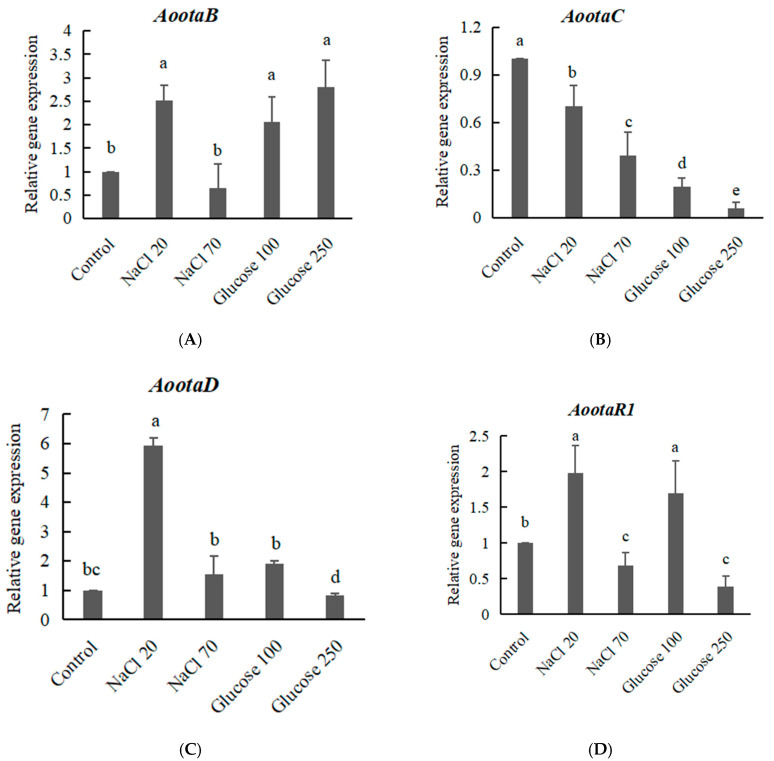
The relative expression level of OTA biosynthetic genes *AootaB* (**A**), *AootaC* (**B**) and *AootaD* (**C**), and regulatory gene *AootaR1* (**D**) in *A. ochraceus* fc-1 at high osmotic conditions after 5 days of incubation. Different letters indicate a significant difference between the corresponding values (*p* < 0.05).

The expressional profile of biosynthetic genes (*otaA-D*) and regulatory gene (*otaR1*) was different under different osmotic stress. Except the *AootaC* gene, the expression level of *AootaA*, *AootaB*, *AootaD* and *AootaR1* was increased 10.5, 2.5, 5.9, 2.0 times higher in *A. ochraceus* at mediun supplied with 20 g/L NaCl than that of the control group without NaCl (Figure 5A and Figure 6B–D). When 70 g/L NaCl was added, the expression level of *AootaA*, *AootaB*, *AootaC* and *AootaR1* was 0.4, 0.6, 0.4 and 0.7 times lower than that of the control group without NaCl (Figure 5A and Figure 6A,B,D). The expression level of *AootaD* was still 1.5 times higher than that of the control group (Figure 6C).

With the addition of 100 g/L, 250 g/L glucose, the expression level of *AootaB* was 2.1 and 2.8 times higher than that of the control group without glucose, respectively (Figure 6A), but the expression level of *AootaC* was 0.2 and 0.06 times lower than that of the control group without glucose, respectively (Figure 6B). The expression level of *AootaA*, *AootaD* and *AootaR1* was increased 1.5, 1.0, and 1.3 times higher in *A. ochraceus* at mediun supplied with 100 g/L glucose than that of the control group without glucose (Figure 5B and Figure 6C,D), and decreased 0.7, 0.8, and 0.4 times lower in *A. ochraceus* at mediun supplied with 250 g/L glucose than that of the control group without glucose (Figure 5B and Figure 6C,D).

## 3. Discussion

Different from other mycotoxins (aflatoxin, fumonisin, deoxynivalenol, zearalenone), ochratoxins are mainly produced by some species in *Aspergillus* and *Penicillium*. Until now, more than 20 species of toxigenic fungi are found to produce ochratoxins, which contaminate different foodstuffs depending on different temperature, humidity and other climatic conditions [2]. OTA could not only be detected in high sugar and low pH-fruits, such as grape and berry [6,7], but also in NaCl and protein rich dry-cured meats and cheeses [12,16,17]. In 2012, the positive rate of OTA in dried vine fruits collected in a Chinese market was 58.9%, with a mean level of 0.99 μg/kg [18]. In 2016, of 30 commercial samples of dried vine fruits analyzed, 10 were contaminated with ochratoxins, and OTA-related metabolites ochratoxin alpha (OTα), ochratoxin B (OTB) and mellein were also detected in different samples [19]. In 2018, the positive rate of OTA in 172 salami samples collected in four regions of Italy was 12.8% [20]. Mycotoxins are secondary metabolites of fungi, and their biological functions are related to the adaptation of fungi to the survival environment (food medium) [21], just like OTA is supportive for growth in high salt environments in *Penicillium* spp., but the biological function of OTA is unclear in *Aspergillus* spp. [15,22].

In fungi, the biosynthesis of ochratoxin A plays an adaptive role in certain food environments. There is a need to understand the mechanism of adaptation of toxigenic fungal species, which are able to colonize highly specialized foods such as cured meats, where there is a high osmotic stress due to the presence of up to 20–22% NaCl during the ripening process [23]. *A. ochraceus* fc-1 and *P. nordicum* 13080 were likely to have greater growth rates in medium supplied with 40 g/L NaCl, compared with no NaCl addition. When 80 g/L NaCl was added, it would always slightly promote the growth of mycelium. Different from mycelium growth, both fungi were likely to produce high levels of OTA in medium supplied with 20 g/L NaCl. In particular, *P. nordicum* 13080 was more suitable to grow in NaCl concentrated medium, and the capability of OTA production was stronger than *A. ochraceus* fc-1; the OTA production was increased to 316 ppb from 77 ppb when 20 g/L NaCl was added. *P. nordicum* is able to successfully adapt to the characteristic NaCl-rich environment of the dry-cured meat products, and OTA is frequently detected in dry-cured meat products [10,20]. Due to different percentages of NaCl added in ripening processing technology, the period of logarithmic growth and OTA yield varies among different fungi in sausage products [24]. Both fungi were more tolerant at moderate ionic a_w_ conditions (0.93 to 0.99) under 28 °C, and the growth of fungi were promoted in medium supplied with certain concentrations of NaCl. Nevertheless, the ionic a_w_ circumstances of production of OTA were very different from those for growth. Different OTA production profiles between the two OTA-producing species were found. OTA biosynthesis gene cluster is the same among these OTA-producing fungi, while the adaptability of the strains to ionic concentration, and the selectivity of strains to host or carbon source are different. The OTA contamination by *P. nordicum* in ham-based media is affected by ionic a_w_ (NaCl), a moderate correlation is found between OTA biosynthetic genes and OTA yield and the activation of biosynthetic genes of *P. nordicum* is earlier than OTA detection [23].

The temporal changes in the expression of the *OtaA* gene by *A. ochraceus* and *P. nordicum* are related to OTA production on medium supplied with certain concentrations of NaCl. The *OtaA* gene was overexpressed by both fungi in medium supplied with 20 g/L NaCl, especially in *P. nordicum*. Gene expression was higher in *P. nordicum* than in *A. ochraceus* in medium supplied with 20 g/L NaCl (Figure 5A). The high significant correlation was found between the early relative expression of the *OtaA* (*otapks*) gene and OTA production in *P. nordicum* [25]. Low-concentration NaCl could promote the expression of the OTA biosynthetic genes, while high-concentration NaCl could inhibit gene expression. The chloride ion is related to the substitution reaction catalyzed by halogenase OtaD in the OTA biosynthesis pathway [13,14], and the expression level of the *AootaD* gene is still up-regulated in high-salt medium (Figure 6C).

Except the NaCl-rich food environment, most *aspergilli* can be isolated from a wide range of products, including cereals, cocoa, coffee, dried fruits, raisins, wine, etc [26]. In particular, *A. carbonarius* are considered as the main source of OTA contamination in grapes, figs, berries and related juice products [27]. The optimum values for growth of *A. carbonarius* on a grape juice-based medium were observed at 30–35 °C and 0.96 a_w_, while for OTA production at 20 °C and 0.98 a_w_ [26]. The sugar content of wine grapes is between 22% and 30%, while eating grapes may be closer to 10–15%. The sugar content of raisins is very high, up to 10~30%, and glucose is predominant. The sugar content of grape juice is about 200 g/L. At the drying stage, grapes are dried until they reach the desired sugar content, around 300 g/L, and a_w_ values decrease from 0.95 to 0.75 a_w_ [28,29]. As a result of the increase in black *aspergilli* during this sun-dried period, high levels of OTA are present in wines produced from dehydrated grapes [29]. As the glucose concentration increases to the highest concentration (250 g/L glucose), there is a tendency for the growth rate to increase in both species, *A. ochraceus* and *A. carbonarius*. OTA production is promoted with added glucose and a certain concentration of glucose (100 g/L) induces significant OTA production in *A. carbonarius* (Figure 4D). Glucose can not promote OTA production in *A. ochraceus*, on the contrary, OTA production is inhibited under high glucose concentration (150 g/L); as a result, the a_w_ values decrease to 0.96 (Figure 4B). The expression of the *OtaA* (*AcOTApks*) was monitored at the same time points along with fungal biomass and OTA accumulation in *A. carbonarius* 5010; the phenomenon was consistent with *P. nordicum*, activation of the biosynthetic genes *OtaA* that was observed a few days before ochratoxin A could be detected [30]. The *otaA* gene was overexpressed by both fungi in medium supplied with 100 g/L glucose, especially in *A. carbonarius* (Figure 5B). The expression of *AootaC* was down-regulated; that could be one reason that the OTA accumulation in *A. ochraceus* supplied with glucose was not increased.

## 4. Conclusions

The results of this study showed the relationship between salt/sugar content and fungal colonization and OTA accumulation. The most powerful strategy to control OTA contamination in cereal, grape related products and dry-cured meat products is the prevention of mycotoxigenic. The mycelium growth was inhibited significantly when salt content reached 100 g/L, and the capacity of sporulation was decreased enormously when salt content was greater than 60 g/L and 80 g/L in *A. ochraceus* and *P. nordicum*. Meanwhile, OTA production was inhibited when salt content was greater than 40 g/L and 60 g/L in *A. ochraceus* and *P. nordicum*, respectively. The addition of glucose (until 250 g/L) could not inhibit mycelium growth and sporulation, and OTA production was inhibited when glucose content was greater than 150 g/L and 200 g/L in *A. ochraceus* and *A. carbonarius*, respectively. The genes involved in the OTA biosynthetic pathway and the positive and negative effects of the osmolytes on their expressions are summarized in Table 1. By controlling NaCl addition, the content of glucose concentration and monitoring the expression of toxigenic genes, the colonization and toxin production was remarkably reduced.

## 5. Materials and Methods

### 5.1. Strains and Media

*A. ochraceus* fc-1 was isolated and characterized in our laboratory [13]. *A. carbonarius* 5010 was given by Prof. Dr. Angelo Visconti and Dr. Giancarlo Perrone. *P. nordicum* 13080 was obtained from the Institute of Sciences of Food Production “Agro-Food Microbial Culture Collection—ITEM”, Italy. These strains were routinely cultured on Potato Dextrose Agar (PDA, Potato 200 g/L; glucose 20 g/L; agar 20 g/L) for 7 days at 28 °C in dark conditions. Conidia from cultures were obtained by scraping them from PDA plates with a sterile cotton swab, and resuspended in sterile water. The conidia count was adjusted to 10^7^ conidia/mL using a haematocytometer. The strains were maintained as conidial suspensions and stored at −80 °C with 15% glycerol and new cultures were used for each experiment [31].

### 5.2. Culture Media Preparation and Culture Conditions

Concentrations of 0, 20, 40, 60, 80, 100 g/L NaCl or 0, 50, 100, 150, 200, 250 g/L glucose were added to PDA medium, and the culture media were prepared by autoclaving for 20 min at 121 °C. *A. ochraceus* fc-1 was cultured at 28 °C for 7 days. *A. carbonarius* 5010 was grown on Yeast Extract Sucrose Agar (YES, yeast extract 20 g/L, sucrose 150 g/L, agar 15 g/L) supplemented with different amounts of glucose (0, 50, 100, 150, 200, 250 g/L), cultured at 28 °C for 7 days; *P. nordicum* 13080 was grown on Malt Extract Agar (MEA, malt extract 30 g/L, soy peptone 3 g/L, agar 15 g/L) medium supplemented with different amounts of NaCl (0, 20, 40, 60, 80, 100 g/L), cultured at 28 °C for 7 days.

### 5.3. Mycelial Growth Rate and Conidia Count

The mycelial growth of *A. ochraceus* fc-1 was evaluated according to [15], with modifications. For analyses of the diameters of the colonies, 5 μL of the 10^7^ conidia mL^−1^ suspension was added dropwise to the center of the solid medium. The colony growth diameter was measured every 12 h using a cross method. Data were analysed using a primary model by plotting colony diameter against time. Data plots showed, after a lag phase, a linear trend with time. The linear part of this graph (linear phase) was used to calculate growth rate (mm/day) [10]. All experiments were done with three replicates per treatment and repeated twice.

The percentage of mycelial growth inhibition was calculated according to the following formula: Inhibition (%) = [(C − T)/C] × 100%, where C is the mean colony diameter for the controls and T is the mean colony diameter for each group treated with NaCl or glucose.

Five agar plugs (diameter 8 mm) were taken from the colony, transferred into 10 mL micro reaction tubes, and 5 mL of sterile water containing 0.01% Tween 80 was added. The spore were dissolved for 2 h at room temperature on a rotary shaker and the amount of conidia was counted using a haemocytometer (Fisher Scientific, Loughborough, UK). All experiments were done with three replicates per treatment and repeated twice.

### 5.4. Extraction and Quantification of OTA

For determination of Ochratoxin A production, the strains were grown at 28 °C on NaCl or glucose supplemented agar plates. Five agar plugs (diameter 8 mm) were taken from the colony, transferred into 2 mL micro reaction tubes and 1 mL of methanol was added. The fungal mycelia were extracted for 2 h at room temperature on a rotary shaker; the mycelia were discarded and the supernatant were filtered through a 0.22 μm filter into the brown vials [31].

The HPLC equipment consisted of an Agilent 1260 series system (Agilent, Berks., UK) with a fluorescence detector and an autosampler. Analysis was done in the isocratic mode and the mobile phase was acetonitrile: water: acetic acid (99:99:2 *v*/*v*/*v*) at a flow rate of 1 mL/min. The injection volume was 20 μL. FLD detection was performed at an excitation wavelength of 330 nm and an emission wavelength of 460 nm, using a C18 column (150 mm × 4.6 mm, 5 μm). Pure ochratoxin A (Sigma, St. Louis, LA, USA) was used as standard [31].

### 5.5. Gene Expression Studies

In order to determine the expression of OTA biosynthesis, genes under different high osmotic conditions, *A. ochraceus* fc-1, *A. carbonarius* 5010 and *P. nordicum* 13080, were cultivated in NaCl- or glucose-supplied media for 5 days at 28 °C in dark. The mycelium used for this assay was collected, dipped in liquid nitrogen immediately for pre-cooling and stored at −80 °C before use. Total RNA was extracted using the RNeasy Plant Mini Kit (QIAGEN, Germany). An amount of 1.0 g of the mycelium was ground in a mortar in the presence of liquid nitrogen. RNA concentration was determined by a Nanodrop Lite spectrophotometer (Thermo Scientific, Waltham, USA). Reverse Transcription PCR (RT-qPCR) was carried out by using the RNA PCR Kit (AMV) (TAKARA, Otsu, Shiga, Japan) [31]. Specific primers used for RT-qPCR were listed in Table 2. The RT-qPCR was reacted using SYBR Green PCR Master Mix and performed in a 7500 Real-Time PCR system (Applied Biosystems, Foster City, CA, USA). The thermal protocol was conducted as described previously [13]. The relative gene expression was calculated with the internal control, and the relative quantification of the target gene expression was calculated according to the method of 2^−ΔΔCT^, where ΔΔC_T_ = (C_T, Target_ − C_T, Ref_)_Treatment_ − (C_T, Target_ − C_T, Ref_)_Control_.

### 5.6. Data Analysis

All the statistical analyses were performed by Microsoft Excel 2013 and SPSS Statistics 21.0. The gene expression analyses were evaluated using one-way analysis of variance (ANOVA). Mean comparison was analyzed through Duncan’s multiple-range test. Differences were considered to be significant at *p* < 0.05. Pearson’s correlation analysis between water activity and ion concentration was performed by SPSS Statistics 21.0 (IBM, Armonk, NY, USA). Pearson’s correlation coefficient (R^2^) was used to assess the correlation between water activity and ion concentration.

## Figures and Tables

**Table 1:** The positive and negative effects of osmotic pressure on OTA biosynthetic genes expression.

Genes	Functions	*P. nordicum*		*A. ochraceus*				*A. carbonarius*	
		NaCl (g/L)		NaCl (g/L)		Glc (g/L)		Glc (g/L)	
		20	70	20	70	100	250	100	250
*otaA*	type i iterative polyketide synthase (PKS)	+	N	+	−	+	N	+	−
*otaB*	nonribosomal peptide synthase (NRPS)			+	N	+	+		
*otaC*	cytochrome p450 monooxygenase			−	−	−	−		
*otaD*	halogenase			+	+	+	−		
*otaR1*	bZIP transcription factor			+	−	+	−		

Note: “+” indicates up regulated significantly, “−” indicates down regulated significantly, “N” indicates no significant change.

**Table 2:** Nucleotide sequences of primers for real time PCR assays.

Primer Name	Sequence (5′ to 3′)	Strain
*GADPH*-F	TGCTCAAGTACGACAGCACC	*Aspergillus ochraceus* [13]
*GADPH*-R	CTCGGCGAAGAACTGAACCT	
*AootaA*-F	CGCCTCATCATCAATCCTT	
*AootaA*-R	CAACTCGGTCAAGCAGAT	
*AootaB*-F	ATACCACCAGAGCTCCAAA	
*AootaB*-R	GAGATGTTCGGTCTGTTCA	
*AootaC*-F	CTTAATACGGTGGTCTACGA	
*AootaC*-R	GAATGATAGGTCCGTATTTCT	
*AootaD*-F	CTATCCGGTGGTCTGTCAGC	
*AootaD*-R	TGAATGCATCGTCGAACCCA	
*AootaR1*-F	GCTTTCAAATCGAATGATTCC	
*AootaR1*-R	GATCGGTTGGAAGTGTAGAA	
*β-tub* F	GCCAGCGGTGACAAGTACGT	*Penicillium nordicum* [10]
*β-tub* R	TACCGGGCTCCAAATCGA	
*PnotaA*-F	CGCCGCTGCGGTTACT	
*PnotaA*-R	GGTAACAATCAACGCTCCCTCTT	
*β-tub* F	CAAACCGGCCAGTGTGGTA	*Aspergillus carbonarius* [32]
*β-tub* R	CGGAGGTGCCATTGTAAACA	
*AcotaA*-F	CGTGTCCGATACTGTCTGTGA	
*AcotaA*-R	GCATGGAGTCCTCAAGAACC	
